# Successful treatment of acute promyelocytic leukemia in a patient under hemodialysis with arsenic trioxide

**DOI:** 10.1002/ccr3.4417

**Published:** 2021-07-23

**Authors:** Akiko Hashimoto, Yasuhiro Tanaka, Takayuki Ishikawa, Isaku Shinzato

**Affiliations:** ^1^ Department of Hematology and Clinical Immunology Kobe City Nishi‐Kobe Medical Center Kobe Japan; ^2^ Department of Hematology Kobe City Hospital Organization Kobe City Medical Center General Hospital Kobe Japan

**Keywords:** acute promyelocytic leukemia, ATO, chronic kidney disease

## Abstract

A man with chronic kidney disease (CKD) under hemodialysis was diagnosed with acute promyelocytic leukemia (APL). He received arsenic trioxide as a single agent and achieved complete molecular remission without severe adverse events. Arsenic trioxide (ATO) can be used safely and effectively for APL with CKD.

## Graphical abstract

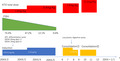

## 1 INTRODUCTION

Acute promyelocytic leukemia (APL) is characterized by a bleeding tendency due to disseminated intravascular coagulation, pancytopenia, and the presence of t(15;17)(q22;q21), resulting in *PML/RARA* fusion gene. All‐trans retinoic acid (ATRA) induces both differentiation and apoptosis of APL cells, and ATRA is the standard treatment for APL. Although ATRA is associated with a high rate of complete remission, up to 20% of patients with APL still relapse. Arsenic trioxide (ATO) was the alternative treatment for relapsed APL.

Currently, no standard strategy for APL complicated with organ failure has been established. In cases complicated with chronic kidney disease (CKD), ATRA was prohibited for APL under hemodialysis in Japan owing to the risk of hypervitaminosis A. Here, we report the case of the APL patient with CKD treated with ATO as a single agent. He achieved molecular complete remission (CR) with ATO. Thus, we consider that this is one of the new strategy for APL treatment in patients undergoing hemodialysis.

## 2 CASE PRESENTATION

**FIGURE 1:**
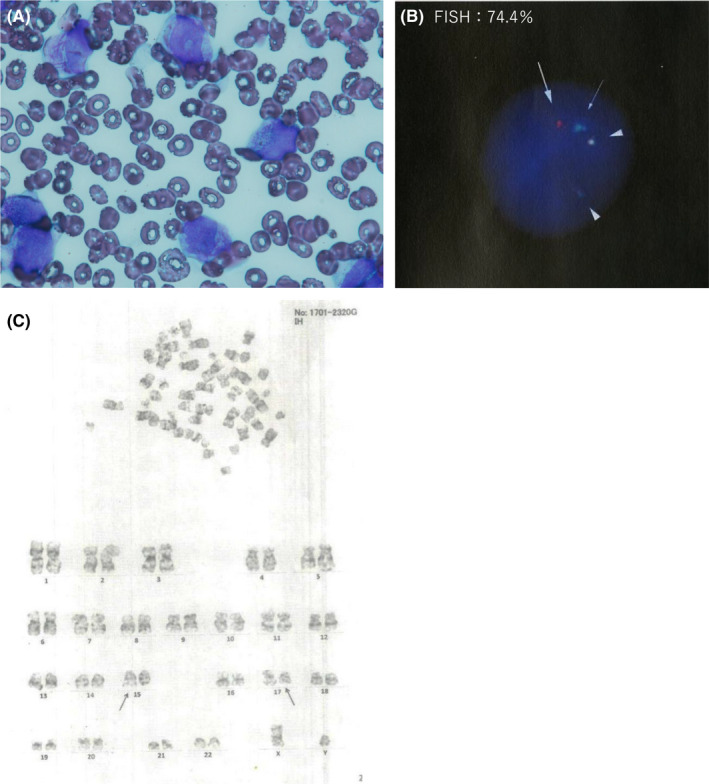
APL at diagnosis. (A) bone marrow smear, (B) FISH (Fluoresence in situ hybridization) on metaphase spreads and interphase nuclei of bone marrow cells, a signal of PML(15q22) probe (arrow), a signal of RARA(17q21) probe(thin arrow), two signals of PML/RARA probe (arrowhead)

A 53‐year‐old man was admitted to another hospital because of pancytopenia. He had been on continuous hemodialysis for 5 years for chronic kidney disease (CKD) that had developed because of polycystic kidney disease. Pancytopenia was detected on routine examination at hemodialysis. On admission, he was asymptomatic. His laboratory data showed that white blood cell; 1000/μL, including 0.5% of promyelocytes with Auer body. Red blood cell count was 3.27 × 10^6^/μL, hemoglobin level was 10.5 g/dL, platelet count was 12.6 × 10^4^/μL, C‐reactive protein level was 0.09 mg/dL, lactate dehydrogenase level was 147 IU/L, blood urea nitrogen level was 32 mg/dL, and creatinine level was 9.64 mg/dL. Bone marrow examination showed that many abnormal promyelocytes were found in the smear specimen (Figure 1A). Fluorescence in situ hybridization (FISH) analysis showed 74.4% of fusion signals between *PML* and *RARA* probe (Figure 1B). Reverse transcriptase‐polymerase chain reaction (RT‐PCR) confirmed the presence of *PML*‐*RARA* fusion transcript. Chromosomal analysis using G‐banding showed 46,XY,t(15;17)(q22;q21)[8]/46,XY[12]. Thus, he was diagnosed with acute promyelocytic leukemia (APL). He was categorized as low risk as per the PETHEMA (Programa para el Estudio de la Terapeutica en Hemopatia maligna) criteria. ATRA was prohibited for hemodialysis‐dependent CKD patients in Japan; therefore, he was given induction therapy that consisted of intravenous arsenic trioxide (ATO) 0.1 mg/kg after hemodialysis every other day. He had end‐stage renal disease and had been undergoing intermittent infusion hemodiafiltration since 2015. Vascular access is venous‐arterial fistula on the left side, dialyzer is used as Toraylight HDF membrane area is 2.1 m^2^, blood flow is 250 mL per minutes, and dialysate flow is 500 mL per minute. The anticoagulant agent was heparin; 1250 units bolus, and 750 units per hour were administered.

**FIGURE 2:**
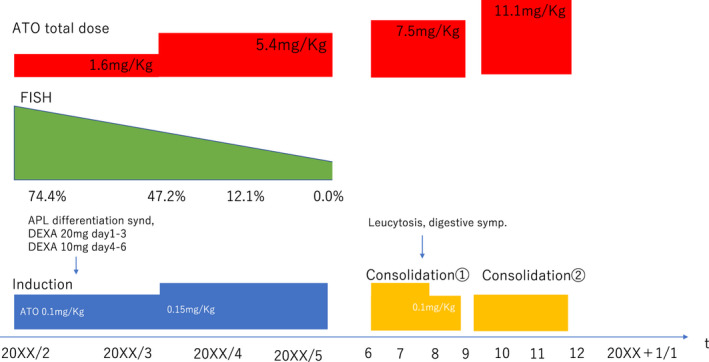
Clinical course

Serum potassium and magnesium levels were maintained at >4.0 mmol/L and 2.0 mEq/L, respectively. The QTc duration was monitored using electrocardiography twice every week. Two weeks after the induction therapy, he developed symptoms of APL differentiation syndrome, such as fever, mild hypoxia, and hyperleukocytosis. His body temperature and hypoxia were reduced with the administration of dexamethasone; 20 mg/d dexamethasone was given for 3 days and 10 mg/d dexamethasone was given for 3 days. ATO was administered for 4 weeks (total dose of 110 mg/body), and he achieved hematological complete response. However, FISH analysis detected 47.2% of fusion signal between *PML* and *RARA* probe in his bone marrow, suggesting that he did not achieve cytogenetic remission. Then, we decided to escalate the dose of ATO at 0.15 mg/kg every other day. After 3 months of induction therapy (total dose of 357 mg/body), bone marrow examination showed 0.3% of promyelocytes in his smear specimen; chromosomal analysis using G‐banding was 46, XY[20/20]; no fusion signal between *PML* and *RARA* probe was detected with FISH analysis; and no fusion transcript was detected by RT‐PCR. These results suggested that he achieved molecular complete remission. During ATO administration, his electrolytes and QTc duration were kept stable using electrocardiography; however, he complained of intermittent gastrointestinal symptoms, such as abdominal distension and pain. Thus, we considered that these symptoms were adverse effects attributed to the administration of ATO, and we reduced the ATO dose to 0.1 mg/kg from 0.15 mg/kg during the first consolidation therapy (total dose of 495 mg/body). After dose reduction in ATO, his gastrointestinal symptoms resolved, and the effect of ATO was maintained. Therefore, we continued to administer 0.1 mg/kg of ATO. After the first consolidation therapy, he received the second consolidation therapy after an interval of 1 month and the third consolidation after an interval of 1 year (Figure 2). At the time of writing this manuscript, he had maintained complete molecular remission for more than 2 years (Table 1).

**TABLE 1:** Treatment of low risk acute promyelocytic leukemia

Reference	Year	Induction therapy	Consolidation therapy and maintenance therapy	Outcome
NCCN guideline^3^	2021	0.15 mg/kg/d of Arsenic trioxide +45 mg/m^2^/d of ATRA until CR or until 60 times of arsenic trioxide administration	Arsenic trioxide 0.15 mg/kg 5/wk for 4 wk every 8 wk for total 4 cycles +ATRA 45 mg/m^2^/d for 2 wk every 4 wk for 7 cycles	‐
Aznab M^17^	2017	0.15 mg/kg/d of ATO until CR	0.15 mg/kg/d of ATO for 28 days as a consolidation therapy The same dose of ATO was given every 3‐4 mo for 14 days for 2 y as a maintenance therapy	Mean DFS 101 and 97 mo (male and female)
Yamamoto et al^14^	2009	ATRA 70 mg/body + 240 mg/m^2^ of behenoyle AraC for 5 days and 30 mg/m^2^ of Daunorubicin	0.15 mg/kg/d of ATO	‐
Vikram et al^11^	2006	0.15 mg/kg/d of ATO until CR	0.15 mg/kg/d of ATO for 4 wk as a consolidation therapy 0.15 mg/kg/d of ATO for 10 d a month for 6 mo as a maintenance therapy	3‐y EFS 74.87% ± 5.6% 3‐y DFS 87.21% ± 4.93% 3‐y OS 86.11% ± 4.08%

## 3 DISCUSSION

To the best of our knowledge, this is the first case wherein ATO was used as a single agent for patients with APL under hemodialysis to achieve and maintain molecular CR. Patients with APL may experience life‐threatening complication early at diagnosis; however, patients who survive generally have very favorable prognosis with the use of ATO and ATRA.^1^, ^2^ Currently, there are no standard treatment guidelines for patients with APL under hemodialysis. Although the combination therapy with ATRA is a standard treatment in patients with APL, it is prohibited in Japan because of hypervitaminosis A for APL patients with CKD. In the case of patients with CKD, ATRA may be overdosed because ATRA is known to be metabolized and excreted in a glucuronide form in the bile and urine^3^, ^4^ and ATRA is not removed during hemodialysis. Hypervitaminosis A may be fatal. Some studies have reported CR achievement with the use of ATRA for APL in patients undergoing hemodialysis.^5^, ^6^, ^7^ However, ATRA is not removed with hemodialysis; therefore, the pharmacokinetics of ATRA are not stable in patients with APL undergoing hemodialysis,^8^ and the efficacy of ATRA is uncertain. ATO can be removed with hemodialysis^9^ and normalizes and stabilizes the serum ATO concentration after 6 months.^8^ ATO can be used repeatedly for APL patients with CKD; therefore, we consider that ATO is suitable for APL patients with CKD.

Some studies have reported that ATO as a single agent can achieve a high CR rate in APL. Mathews et al^10^ used ATO in 72 newly diagnosed patients (patients without organ failure) and reported complete hematological remission in 86.1% of the patients. They also reported that the 3‐year Kaplan‐Meier estimate of event‐free survival (EFS), disease‐free survival (DFS), and overall survival (OS) was 74.8%, 87.21%, and 86.11%, respectively, at a median follow‐up of 25 months. Shen et al^11^ described outcomes for ATO‐based treatment in 15 relapsed APL patients: 10 patients achieved CR with the use of ATO as a single agent. In particular, low‐ and intermediate‐risk groups, as per the PETHEMA criteria, tend to achieve more benefits than high‐risk groups.^12^ Our patient was categorized as being at low risk as per the PETHEMA criteria; therefore, we decided to treat our patient using ATO as a single agent. Major adverse events of ATO include APL differentiation syndrome, electrode abnormality, and long QT syndrome. Yamamoto et al^13^ reported some case that APL with hemodialysis can safely use ATO while monitoring plasma arsenic concentrations; they measured the plasma ATO concentration and managed the adverse events. However, we cannot measure the serum ATO concentration at our hospital. Therefore, we regularly check the electrode and electrocardiogram and manage these advert events even when the toxicity of ATO is mild and reversible.

There is one issue that needs to be considered while administering ATO for APL. There are limited reports on the long‐term outcome of using single‐agent ATO in the management APL; further, it is unclear how many sessions of consolidation therapy with ATO are required to treat APL patients with CKD. Lo‐Coco et al^14^ reported that ATRA/ATO combination therapy may be superior to ATRA + IDR as the induction therapy and four sessions of consolidation therapy. The NCCN guideline recommends four sessions of consolidation therapy.^15^ However, there is no consensus regarding the optimal number of consolidation therapy sessions for the treatment of APL with ATO as a single agent. In particular, the case of our patient was complicated with CKD. Emmons et al^16^ reported that an APL patient under hemodialysis achieved CR with ATO as a single agent and maintained CR for 3 years with combination therapy of ATO and idarubicin. Although a conventional dose of ATO is 0.15 mg/kg, they use 0.1 mg/kg of ATO. They mentioned in Discussion Part that as arsenic trioxide concentration were not assessed or monitored, the titration of arsenic trioxide was based on toxicity profile. We decided to start the decreased dose of 0.1 mg/kg and increase to 0.15 mg/kg if adverse event does not occur. This patient was treated with ATO as a single agent for the induction therapy; however, consolidation therapy involved not only ATO, but also idarubicin. Previously, two studies have reported the long‐term outcome of using a single agent, ATO, in APL treatment.^10^, ^17^ Mathews et al reported the efficacy and minimal toxicity of ATO in a newly diagnosed APL patient with the following regimen. ATO was administered at 10 mg daily dose for adults and 0.15 mg/kg for pediatric patients until CR was achieved. Another 4‐week course was administered after a 4‐week interval as consolidation therapy to those who had achieved CR. Subsequently, after a second 4‐week interval, it was administered for 10 d/mo for 6 months as maintenance therapy. Mozaffar Aznab and Mabdour Rezzael reported the result of induction, consolidation, and maintenance therapies with ATO as a single agent in an 11‐year follow‐up. They reported that ATO was infused at a daily dose of 0.15 mg/kg as induction therapy until CR was achieved. Following 2 week of rest, ATO was administered daily for 28 days as consolidation therapy. Then, ATO was administered for 14 days every 3‐4 months for 2 years. Both the reports were different with respect to the method of consolidation and maintenance therapy; however, they reported a CR rate and long‐term survival rate of >80%. Further research on a larger sample is necessary to establish the method of consolidation and maintain therapy with ATO as a single agent.

In summary, single‐agent ATO is useful for treating patients with APL under hemodialysis to achieve and maintain molecular CR. ATO can be used safely with careful monitoring of electrolytes and electrocardiography without measuring the serum ATO concentration. Further research involving more number of similar cases is required to verify the appropriate number of consolidation therapy sessions.

## CONFLICT OF INTEREST

None declared.

## AUTHOR CONTRIBUTIONS

AH: designed this project and wrote the manuscript. AH, YT, and IS: managed the patient. YT and TI: supervised this project and critically revised the manuscript. All authors: approved the final version of the manuscript.
